# Treatment of Rickets

**Published:** 1891-05

**Authors:** 


TREATMENT OF RICKETS.—The treatment of rickets should be by food, rather than by drugs. Raw meat is of more value than iron, and cream or fresh milk than cod liver oil. The diet must be carefully examined to see that it contains a due portion of fat, proteids and salts. A sufficiently close estimate is easily made since the composition of milk and of all food used for children is accurately known. The amount of animal fat in a rickety child's food must equal at least one-fourth of the total solids taken; proteids and carbo-hydrates about one-third, and salts, about one-tenth. Such a diet will cure rickets without drugs. Iron is often a useful adjunct. The salts of lime may be added in the form of lacto-phosphate. Potent aids are sunlight, fresh air and warm clothing.—*Indiana Med. Journal.*

